# Supplementary material for: The endoplasmic reticulum–plasma membrane tethering protein TMEM24 is a regulator of cellular Ca2+ homeostasis
Source: J Cell Sci. 2021 Dec 16;135(5):jcs259073. doi: 10.1242/jcs.259073 (PMC8729788; doi:10.1242/jcs.259073)
Supplement: Supplementary information [file joces-135-259073-s1.pdf]

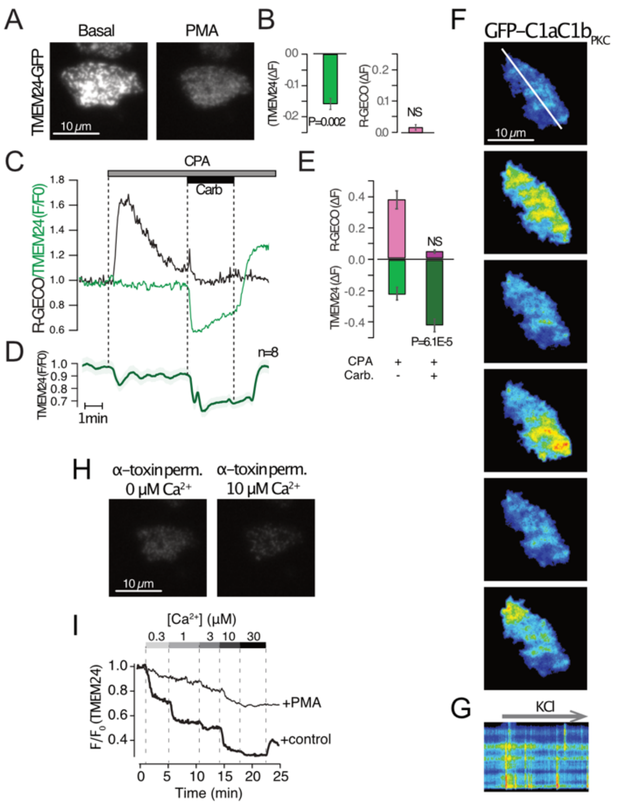

Fig. S1.

(A) TIRF microscopy images GFP-TMEM24 fluorescence from a MIN6 cell exposed to 1 µM of the DAG analogue PMA.

(B) Quantifications of the PMA-induced dissociation of GFP-TMEM24 from the plasma membrane and the corresponding R-GECO fluorescence change in the same cells (means±SEM for 14 cells).

(C) TIRF microscopy recording of R-GECO (black; Ca^{2+}) and GFP-TMEM24 (green) fluorescence from a MIN6 cells exposed to 100 µM CPA and 10 µM carbachol.

(D) Average GFP-TMEM24 fluorescence change from 6 cells on one coverslip.

(E) Quantifications of the CPA- and charbachol-induced changes in GFP-TMEM24 plasma membrane fluorescence and the corresponding R-GECO fluorescence change in the same cells (means±SEM for 65 cells).

(F, G) TIRF microscopy images from a MIN6 cell expressing the DAG-sensor GFP-C1aC1b$_{PKC}$ during depolarization with 30 mM KCl. Notice the irregular formation of DAG at the plasma membrane, apparent as transient, localized fluorescence increase events in the kymograph in D.

(H) TIRF microscopy images GFP-TMEM24 fluorescence from an α-toxin-permeabilized MIN6 cell pre-incubated with 1 µM of the DAG analogue PMA and exposed to the indicated Ca^{2+} buffer.

(I) TIRF microscopy recordings of GFP-TMEM24 fluorescence from two representative α-toxin-permeabilized MIN6 cells exposed (grey) or not (black) to 1 µM PMA. Notice that preincubation with PMA leads to reduced Ca^{2+}-induced GFP-TMEM24 plasma membrane dissociation.

Journal of Cell Science • Supplementary information

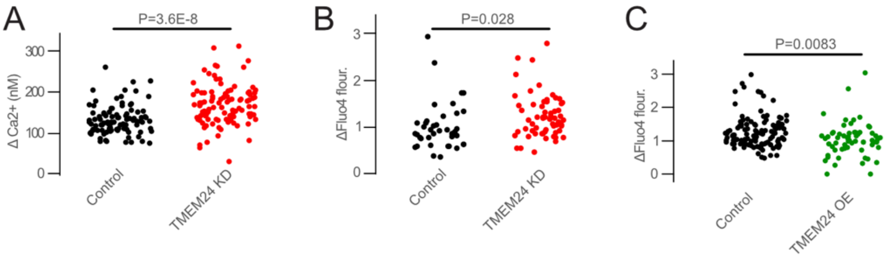

Fig. S2.

(A) Ca^{2+} concentration change in response to 100 µM CPA in MIN6 cells treated for 48h with control siRNA (black) or siRNA against TMEM24 (red). Ca^{2+} measurements were done with Fura-2.

(B) Ca^{2+} concentration change in response to 10 µM carbachol in MIN6 cells treated for 48h with control siRNA (black) or siRNA against TMEM24 (red). Ca^{2+} measurements were done with Fluo-4.

(C) Ca^{2+} concentration change in response to 100 µM CPA in MIN6 cells over-expressing mCherry (control; black) or mCherry-TMEM24 (TMEM24 OE; green). Ca^{2+} measurements were done with Fluo-4.

Journal of Cell Science • Supplementary information

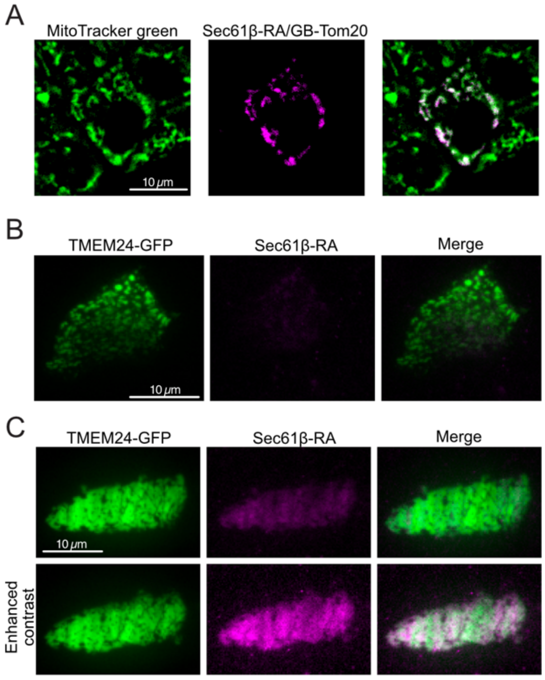

Fig. S3.

(A) Confocal microscopy images of MIN6 cells expressing the ER-mitochondria contact site reporter (Sec61b-RA+ GB-Tom20; magenta) and loaded with MitoTracker green to label mitochondria (green).

(B) TIRF microscopy images of a MIN6 cell expressing TMEM24-GFP (green) and Sec61b-RA. Notice the very weak fluorescence from Sec61b-RA in the absence of any GB expression.

(C) TIRF microscopy images of a MIN6 cell expressing TMEM24-GFP (green) and Sec61b-RA. Notice that RA fluorescence is very dim in the absence of GB expression, but that contrast enhancement reveals RA fluorescence that overlaps with that of TMEM24-GFP, consistent with ER-localization.

Journal of Cell Science • Supplementary information
