# Supplementary material for: The endoplasmic reticulum–plasma membrane tethering protein TMEM24 is a regulator of cellular Ca2+ homeostasis
Source: J Cell Sci. 2021 Dec 16;135(5):jcs259073. doi: 10.1242/jcs.259073 (PMC8729788; doi:10.1242/jcs.259073)
Supplement: Reviewer comments [file joces-135-259073_review_history.pdf]

The endoplasmic reticulum-plasma membrane tethering protein TMEM24 is a regulator of cellular Ca^{2+} homeostasis

Beichen Xie, Styliani Panagiotou, Jing Cen, Patrick Gilon, Peter Bergsten and Olof Idevall-Hagren

DOI: 10.1242/jcs.259073

Editor: James Olzmann

Review timeline

Original submission:	27 June 2021
Editorial decision:	16 August 2021
First revision received:	10 November 2021
Accepted:	13 November 2021

Original submission

First decision letter

MS ID#: JOCES/2021/259073

MS TITLE: The endoplasmic reticulum-plasma membrane tethering protein TMEM24 is a regulator of cellular Ca2+ homeostasis

AUTHORS: Beichen Xie, Styliani Panagiotou, Jing Cen, Patrick Gilon, Peter Bergsten, and Olof Idevall-Hagren
ARTICLE TYPE: Research Article

We have now reached a decision on the above manuscript.

To see the reviewers' reports and a copy of this decision letter, please go to: https://submit-jcs.biologists.org and click on the 'Manuscripts with Decisions' queue in the Author Area. (Corresponding author only has access to reviews.)

As you will see, both reviewers indicate that the manuscript is well conducted and interesting. However, they raise a number of criticisms that prevent me from accepting the paper at this stage. They suggest, however, that a revised version might prove acceptable, if you can address their concerns. If you think that you can deal satisfactorily with the criticisms on revision, I would be pleased to see a revised manuscript. We would then return it to the reviewers.

We are aware that you may be experiencing disruption to the normal running of your lab that makes experimental revisions challenging. If it would be helpful, we encourage you to contact us to discuss your revision in greater detail. Please send us a point-by-point response indicating where you are able to address concerns raised (either experimentally or by changes to the text) and where you will not be able to do so within the normal timeframe of a revision. We will then provide further guidance. Please also note that we are happy to extend revision timeframes as necessary.

Please ensure that you clearly highlight all changes made in the revised manuscript. Please avoid using 'Tracked changes' in Word files as these are lost in PDF conversion.

© 2021. Published by The Company of Biologists under the terms of the Creative Commons Attribution License (https://creativecommons.org/licenses/by/4.0/).

I should be grateful if you would also provide a point-by-point response detailing how you have dealt with the points raised by the reviewers in the 'Response to Reviewers' box. Please attend to all of the reviewers' comments. If you do not agree with any of their criticisms or suggestions please explain clearly why this is so.

Reviewer 1

Advance summary and potential significance to field

This is an interesting manuscript exploring the role of TMEM24, an ER protein dynamically localized at the ER-PM contact sites, on the regulation of intracellular calcium stores and glucose stimulated insulin secretion (GSIS) in mouse pancreatic beta cells. The authors show that TMEM24 is weakly bound to the plasma membrane and that, in agreement with previous reports, its ER-PM localization is negatively regulated by cytosolic calcium. They add on that by showing that TMEM24 presence in the ER-PM is also dependent on diacylglycerol. The authors also show that TMEM24 is potentially localized to the ER-Mitochondria contact sites and regulates calcium homeostasis in these organelles. In contrast with previous reports, the authors present evidence that TMEM24 is dispensable for GSIS in mouse pancreatic beta cells.

The study is well conducted and is worthy of publication after revisions and further explanation concerning the controversial data on the role of TMEM24 in pancreatic beta cells GSIS. Also, the TMEM24 localization at the ER-mitochondria junctions is not properly evaluated and critically tested. The manuscript needs some reorganization to make clear what is new and what is validation of previously publish data.

Comments for the author

Major concerns and suggestions:
1-The authors show that TMEM24 is weakly bound to PM and its localization at ER-PM is regulated by DAG. This is important new information to the field. To further validate these findings in intact cells, I would suggest treating the cells with the DAG analogue PMA in the presence or absence of BAPTA-AM (a calcium chelator) in order to prevent any changes in cytosolic calcium and dissociate these two agents.
2-Contrary to two previous reports (Pottekat, A. et al 2013 and Less, J.et al 2017), it is shown here that TMEM24 is not essential for GSIS in two different cells models (TMEM24 knockdown and knockout MIN6 cells). The reasons for this discrepancy are not clear and should be further evaluated and discussed. In Less J et al 2017, TMEM knockdown in Ins-1 cells leads to significant decrease in GSIS.
Similar results are shown by Pottekat, A. et al 2013 in both Ins1 cells and MIN6 pseudoislets. Have the authors tried to perform the GSIS experiments in Ins-1 cells or MIN6 plated as pseudoislets? I think this should be done to further evaluate if different experimental conditions could explain the discrepant results. Also, this topic should be better discussed in the “Discussion” section.
3- The lowering in cytosolic calcium after high glucose addition in control MIN-6 cells is not convincing. The window is very small and there is no evidence that this could be explained by glucose-induced calcium uptake by SERCA.
4-The authors show that TMEM24 down-regulation results in higher mitochondria calcium accumulation. This is accompanied by decreased mitochondria membrane potential and lower respiration suggesting lower ATP production in cells with absence of TMEM24. Given that ATP is a key component of the regulation of GSIS wouldn’t one expect that these cells would have compromised insulin secretion?
5- Although TMEM24 localization at ER- mitochondria contact sites is an interesting hypothesis the data provided does not allow such a claim. This needs to be properly addressed with super resolution microscopy that goes beyond the diffraction limit of the light (200-300nm resolution of regular confocal microscopy). Alternatively, EM/immunogold or correlative EM (CLEM) could be used.

© 2021. Published by The Company of Biologists under the terms of the Creative Commons Attribution License (https://creativecommons.org/licenses/by/4.0/).

Minor:
1-Page 6, line 10 of main text, (ref) is missing.
2-It's not possible to see mitochondria morphology in fig 4G. Better resolution pictures should be provided.

Reviewer 2

Advance summary and potential significance to field

This manuscript by Xie et al. aims to clarify the role of the ER-plasma membrane (PM) tethering protein TMEM24 in regulating insulin secretion in pancreatic beta-cells. Previous reports (Lees et al. 2017, Sun et al. 2019)
have demonstrated reversible localization of TMEM24 at ER-PM contacts regulated by Ca2+ oscillation and phosphorylation. In addition, Pottekat et al 2013 and Lees et al 2017 showed an important role of TMEM24 in regulating insulin secretion in beta cells. It was proposed that TMEM24 regulates pulsatile Ca2+ increase and insulin secretion by localizing at ER-PM contacts and transporting PI to the PM for PI(4,5)P2 synthesis.
In this manuscript, the authors first showed that TMEM24 dissociation from ER-PM contacts can be induced by a relatively small increase in Ca2+ or by stimuli that increase DAG without elevating Ca2+. Surprisingly, the authors showed that glucose-induced Ca2+ responses and insulin secretion were not significantly affected in TMEM24 KO or knockdown (KD) MIN6 cells. Moreover membrane depolarization by KCl even enhanced Ca2+ responses and insulin secretion in TMEM24 KO and KD MIN6 cells. These results contradict the findings using INS1 cells reported by Lees et al. The authors further showed that ER and mitochondrial Ca2+ levels were elevated in TMEM24 KO cells, which may explain the impaired glucose-induced increase of oxygen consumption in these cells. Lastly, the authors showed that TMEM24 forms clusters near mitochondria after dissociation from ER-PM contacts induced by KCl using fluorescence microscopy. The authors propose that release of TMEM24 into the bulk ER enables its "direct" interactions with mitochondria and loss of TMEM24 causes Ca2+ overload in the ER and mitochondria, impairing mitochondria functions.
Overall, this study showed that TMEM24 is actually not required for glucose-stimulated insulin secretion. The study also provides new findings supporting a role of TMEM24 in regulating ER and mitochondria Ca2+ homeostasis.

Comments for the author

Major concerns:
1. There is no data supporting the authors' claim in the Abstract that TMEM24 directly interacts with mitochondria. Figure 4P showed formation of TMEM24 clusters after KCl treatment which became more pronounced after washout in one MIN6 cell. Nevertheless, the increase in TMEM24 signal in clusters was accompanied with a decrease, instead of increase, of mitochondria signal.
Also, there is a lack of quantification and no N or n numbers were shown. What is the percentage of cells that showed intracellular TMEM24 clusters following dissociation from the PM? Are all TMEM24 clusters observed closely associated with mitochondria? It should be noted that imaging data acquired by light microscopy cannot support the presence of TMEM24 at ER-mitochondria contacts or TMEM24-mitochondria interaction. The claim should be removed if there is a lack of sufficient evidence.
2. The main conclusion of this study is TMEM24 is a regulator of cellular Ca2+ homeostasis. Nevertheless, the data supporting this conclusion were derived only from TMEM24 KO MIN6 cells, and it is unclear how TMEM24 regulates ER and mitochondrial Ca2+ levels. At a minimum, the authors should rule out the possibility that abnormal Ca2+ levels in the ER and mitochondria is not a pleiotropic effect in TMEM24 KO cells. Does re-expression of TMEM24 rescue ER and mitochondria Ca2+ overload in TMEM24 KO MIN6 cells? Also, is ER and mitochondria Ca2+ overload observed in TMEM24 knockdown MIN6 cells?
3. This study claims that TMEM24 is not required for glucose-stimulated insulin secretion which is contrast to previous findings. Are the observed differences caused by the choice of cell lines or different experimental conditions? Another beta cell model should be tested in order to rule out that the finding in this study only applies to MIN6 cells.
Minor concerns:

© 2021. Published by The Company of Biologists under the terms of the Creative Commons Attribution License (https://creativecommons.org/licenses/by/4.0/).

1. In some places, the descriptions in the figure legends and main texts are not consistent. For example: it says in the text that Ca2+ responses were measured using Cal520 but in the legend of Figure 2, Fluo4 was mentioned instead.
2. The figure legends and methods sections did not provide sufficient details. For example, in Figure 3O, the concentration of thapsigargin used was not specified. Also, the amount of time cells were treated with tharsigargin was not described.
3. The manuscript will benefit from careful scientific proof-reading.
There are some mistakes in the text. For example, it says "….electrostatic interactions between the C-terminus and cationic lipids in the plasma membrane". It should be "negatively charged lipids" in the plasma membrane.
4. The authors showed that TMEM24 dissociation from ER-PM contacts can be triggered by carbachol stimulation after ER Ca2+ depletion or PMA addition.
Based on these results, the authors claim that TMEM24 binding to the plasma membrane can be regulated by DAG. The authors did not directly demonstrate the involvement of DAG in regulating TMEM24 dissociation. Thus, the conclusion should be modified accordingly.

First revision

Author response to reviewers' comments

Reviewer 1 Advance Summary and Potential Significance to Field:
This is an interesting manuscript exploring the role of TMEM24, an ER protein dynamically localized at the ER-PM contact sites, on the regulation of intracellular calcium stores and glucose stimulated insulin secretion (GSIS) in mouse pancreatic beta cells. The authors show that TMEM24 is weakly bound to the plasma membrane and that, in agreement with previous reports, its ER-PM localization is negatively regulated by cytosolic calcium. They add on that by showing that TMEM24 presence in the ER-PM is also dependent on diacylglycerol. The authors also show that TMEM24 is potentially localized to the ER-Mitochondria contact sites and regulates calcium homeostasis in these organelles. In contrast with previous reports, the authors present evidence that TMEM24 is dispensable for GSIS in mouse pancreatic beta cells.

The study is well conducted and is worthy of publication after revisions and further explanation concerning the controversial data on the role of TMEM24 in pancreatic beta cells GSIS. Also, the TMEM24 localization at the ER- mitochondria junctions is not properly evaluated and critically tested. The manuscript needs some reorganization to make clear what is new and what is validation of previously publish data.

Reviewer 1 Comments for the Author:
Major concerns and suggestions:

1- The authors show that TMEM24 is weakly bound to PM and its localization at ER-PM is regulated by DAG. This is important new information to the field. To further validate these findings in intact cells, I would suggest treating the cells with the DAG analogue PMA in the presence or absence of BAPTA-AM (a calcium chelator) in order to prevent any changes in cytosolic calcium and dissociate these two agents.

We agree that it is important to confirm the involvement of DAG in the direct regulation of TMEM24 localization. We have now added quantifications that show that the PMA-induced TMEM24 dissociation occurs independent of any Ca2+ concentration change (suppl. Fig. 1B). We have also performed simultaneous recordings of GFP-TMEM24 plasma membrane binding and the intracellular Ca2+ concentration under Ca2+-free conditions (0 mM Ca2+, 2 mM EGTA) following depletion of intracellular Ca2+ stores (with CPA) and elevation of DAG by charbachol-induced activation of PLC. Whereas CPA addition both elevates Ca2+ and cause TMEM24 plasma membrane dissociation, the subsequent addition of carbachol only causes pronounced dissociation of TMEM24 without detectable change in the intracellular Ca2+ concentration (see Suppl. Fig. 1C-E). We therefore believe that we have shown that DAG can induce TMEM24

© 2021. Published by The Company of Biologists under the terms of the Creative Commons Attribution License (https://creativecommons.org/licenses/by/4.0/).

2- Contrary to two previous reports (Pottekat, A. et al 2013 and Less, J. et al 2017), it is shown here that TMEM24 is not essential for GSIS in two different cells models (TMEM24 knockdown and knockout MIN6 cells). The reasons for this discrepancy are not clear and should be further evaluated and discussed. In Less, J et al 2017, TMEM knockdown in Ins-1 cells leads to significant decrease in GSIS. Similar results are shown by Pottekat, A. et al 2013 in both Ins1 cells and MIN6 pseudoislets. Have the authors tried to perform the GSIS experiments in Ins-1 cells or MIN6 plated as pseudoislets? I think this should be done to further evaluate if different experimental conditions could explain the discrepant results. Also, this topic should be better discussed in the "Discussion" section.

According to the reviewers' suggestion we have now performed insulin secretion measurements from wildtype and TMEM24 KO MIN6 pseudo-islets. As can be seen in new Figure 2O, both WT and TMEM24 KO pseudo-islets secrete insulin in response to glucose. We monitored the kinetics of secretion by islet perifusion and found that whereas the initial response (1st phase insulin secretion) is similar in WT and TMEM24 KO islets, the sustained secretion (2nd phase) is slightly impaired in the KO islets. The fact that this impairment was not seen in monolayer cultures may be due either to lack of temporal resolution (we measure bulk secretion during 30 min and may easily miss a small defect in the 2nd phase of secretion) or to the fact that pseudo-islets are more heavily dependent on glucose oxidation and oxidative phosphorylation for normal secretion of insulin. This is now also discussed in the paper (highlighted in yellow in the manuscript text).

3- The lowering in cytosolic calcium after high glucose addition in control MIN-6 cells is not convincing. The window is very small and there is no evidence that this could be explained by glucose-induced calcium uptake by SERCA.

We refer in the text to work done by Roe and Dukes (PMID: 8023914) where they show that acute elevation of the glucose concentration results in the immediate lowering of the cytosolic Ca2+ concentration, and that this lowering is completely prevented when the SERCA inhibitor thapsigargin is present. This observation has been reproduced in many studies since, including studies showing the lack of lowering in SERCA KO beta cells and by direct measurements of the ER Ca2+ concentration (e.g. PMID: 21885870).

4- The authors show that TMEM24 down-regulation results in higher mitochondria calcium accumulation. This is accompanied by decreased mitochondria membrane potential and lower respiration suggesting lower ATP production in cells with absence of TMEM24. Given that ATP is a key component of the regulation of GSIS, wouldn't one expect that these cells would have compromised insulin secretion?

Yes, we agree that impaired glucose-induced ATP production would be expected to impair insulin secretion from the cells. One complicating factor is that the voltage-dependent Ca2+ influx is more pronounced in TMEM24 KO cells. This may partially compensate for the impaired ATP production by triggering the exocytosis of more insulin granules (i.e. also involving granules at longer distance from the voltage- dependent Ca2+ channel clusters in the plasma membrane). Dynamic measurements of insulin secretion from pseudo-islets indeed revealed a small, but significant, time-dependent decrease in insulin secretion that selectively affected the 2nd phase of insulin secretion (Fig. 2O). Similarly, we also saw a small, time- dependent decrease in glucose-induced Ca2+ signals in the KO cells (Fig. 2I). Together, these observations points to a role of TMEM24 in maintaining sustained insulin secretion, likely through regulation of mitochondrial metabolism. This is also more extensively discussed in the manuscript (highlighted in yellow).

5- Although TMEM24 localization at ER- mitochondria contact sites is an interesting hypothesis the data provided does not allow such a claim. This needs to be properly addressed with super resolution microscopy that goes beyond the diffraction limit of the light (200-300nm resolution of regular confocal microscopy). Alternatively, EM/immunogold or correlative EM (CLEM) could be used.

© 2021. Published by The Company of Biologists under the terms of the Creative Commons Attribution License (https://creativecommons.org/licenses/by/4.0/).

We agree with the reviewer that the data we provided in the manuscript does not give strong support to our hypothesis that TMEM24 may operate at ER-mitochondria contact sites. We believe that it would be difficult to observe the brief association between TMEM24 and mitochondria by immunogold EM or CLEM, as it would require rapid fixation within a narrow time window. In an attempt to give some more support to our statement, we have performed the following experiments and analyses:

1) **We have performed simultaneous confocal microscopy imaging of TMEM24-GFP and Tom20-mApple (mitochondria) before, during and after depolarization with 30 mM KCl. We have then threshold segmented the mitochondria to generate a mask of these structures and then determined how TMEM24-GFP fluorescence changes on these structures during depolarization. We find that depolarization is associated with increased TMEM24-GFP localization to mitochondrial structures, and the association is reversed when KCl is washed out (new Fig. 5A,B).**
2) **Since the resolution of our confocal setup is around 250 nm in XY and >500 nm in Z we cannot prove that TMEM24 directly bind to mitochondria. To overcome the resolution limit in Z, we repeated these experiments using TIRF microscopy, where the resolution in the Z-axis is around 80 nm. Although scarce, we do observe TMEM24 colocalization with sub-plasma membrane-localized mitochondria (new Fig. 5C).**
3) **In an attempt to overcome the resolution limitation of our microscope, we used a membrane proximity biosensor based on fluorescent protein exchange (see PMID: 22444590 and 23656278). Briefly, we utilized a red fluorescent protein heterodimer and placed one subunit on the ER surface and the other on the mitochondria surface. When the two proteins are in proximity, they fold into a fluorescent protein detectable by regular fluorescence microscopy. We expressed the two fusion proteins in MIN6 cells and show that they label parts of the ER in proximity of mitochondria and vice versa. Co-expressed TMEM24- GFP accumulated at sites of ER-mitochondria proximity following depolarization (new Fig. 5D-F and Suppl. Fig. 3). These experiments are at least consistent with TMEM24 operating at locations of proximity between the ER and mitochondria.**

In addition to these experiments, we have also made changes the text to soften the statement that TMEM24 act at ER-mitochondria contacts and completely removed this statement from the abstract.

Minor:
1- Page 6, line 10 of main text, (ref) is missing.

Thank you. This has how been corrected.

2- It’s not possible to see mitochondria morphology in fig 4G. Better resolution pictures should be provided.

Images have been replaced with new images of higher resolution.

Reviewer 2 Advance Summary and Potential Significance to Field: This manuscript by Xie et al. aims to clarify the role of the ER-plasma membrane (PM) tethering protein TMEM24 in regulating insulin secretion in pancreatic beta- cells. Previous reports (Lees et al. 2017, Sun et al. 2019) have demonstrated reversible localization of TMEM24 at ER-PM contacts regulated by Ca2+ oscillation and phosphorylation. In addition, Pottekat et al 2013 and Lees et al 2017 showed an important role of TMEM24 in regulating insulin secretion in beta cells. It was proposed that TMEM24 regulates pulsatile Ca2+ increase and insulin secretion by localizing at ER-PM contacts and transporting PI to the PM for PI(4,5)P2 synthesis. In this manuscript, the authors first showed that TMEM24 dissociation from ER-PM contacts can be induced by a relatively small increase in Ca2+ or by stimuli that increase DAG without elevating Ca2+. Surprisingly, the authors showed that glucose-induced Ca2+ responses and insulin secretion were not significantly affected in TMEM24 KO or knockdown (KD) MIN6 cells. Moreover, membrane depolarization by KCl even enhanced Ca2+ responses and insulin secretion in TMEM24 KO and KD MIN6 cells. These results contradict the findings using INS1 cells reported by Lees et al. The authors further showed that ER and mitochondrial Ca2+ levels were elevated in TMEM24 KO cells, which may explain the impaired glucose-induced increase of oxygen consumption in these cells. Lastly, the authors showed that TMEM24 forms clusters near mitochondria after dissociation from ER-PM contacts induced by KCl using fluorescence microscopy. The authors propose that release of TMEM24 into the bulk ER enables its “direct” interactions with

© 2021. Published by The Company of Biologists under the terms of the Creative Commons Attribution License (https://creativecommons.org/licenses/by/4.0/).

mitochondria and loss of TMEM24 causes Ca2+ overload in the ER and mitochondria, impairing mitochondria functions.
Overall, this study showed that TMEM24 is actually not required for glucose-stimulated insulin secretion. The study also provides new findings supporting a role of TMEM24 in regulating ER and mitochondria Ca2+ homeostasis.

Reviewer 2 Comments for the Author:
Major concerns:
1. There is no data supporting the authors' claim in the Abstract that TMEM24 directly interacts with mitochondria. Figure 4P showed formation of TMEM24 clusters after KCl treatment which became more pronounced after washout in one MIN6 cell. Nevertheless, the increase in TMEM24 signal in clusters was accompanied with a decrease, instead of increase, of mitochondria signal. Also, there is a lack of quantification and no N or n numbers were shown. What is the percentage of cells that showed intracellular TMEM24 clusters following dissociation from the PM? Are all TMEM24 clusters observed closely associated with mitochondria? It should be noted that imaging data acquired by light microscopy cannot support the presence of TMEM24 at ER-mitochondria contacts or TMEM24-mitochondria interaction. The claim should be removed if there is a lack of sufficient evidence.

We agree with the reviewer that the data presented in the manuscript do not prove that TMEM24 binds directly to mitochondria. In an attempt to determine if TMEM24 may interact with mitochondria following its release from the plasma membrane, we performed the following experiments:
1) We have performed simultaneous confocal microscopy imaging of TMEM24-GFP and Tom20-mApple (mitochondria) before, during and after depolarization with 30 mM KCl. We have then threshold segmented the mitochondria to generate a mask of these structures and then determined how TMEM24-GFP fluorescence changes on these structures during depolarization. We find that depolarization is associated with increased TMEM24-GFP localization to mitochondrial structures, and the association is reversed when KCl is washed out (new Fig. 5A,B).
2) Since the resolution of our confocal setup is around 250 nm in XY and >500 nm in Z we cannot prove that TMEM24 directly bind to mitochondria. To overcome the resolution limit in Z, we repeated these experiments using TIRF microscopy, where the resolution in the Z-axis is around 80 nm. Although scarce, we do observe TMEM24 colocalization with sub-plasma membrane-localized mitochondria (new Fig. 5C).
3) In an attempt to overcome the resolution limitation of our microscope, we used a membrane proximity biosensor based on fluorescent protein exchange (see PMID: 22444590 and 23656278). Briefly, we utilized a red fluorescent protein heterodimer and placed one subunit on the ER surface and the other on the mitochondria surface. When the two proteins are in proximity, they fold into a fluorescent protein detectable by regular fluorescence microscopy. We expressed the two fusion proteins in MIN6 cells and show that they label parts of the ER in proximity of mitochondria and vice versa. Co-expressed TMEM24- GFP accumulated at sites of ER-mitochondria proximity following depolarization (new Fig. 5D-F and Suppl. Fig. 3). These experiments are at least consistent with TMEM24 operating at locations of proximity between the ER and mitochondria.
Together, we believe that the results from these experiments are consistent with TMEM24 exerting direct effects on mitochondria at ER-mitochondria contact sites, although we are aware that the resolution of imaging setups used precludes us from definitively prove this. We therefore more careful when discussing the putative action of TMEM24 at ER-mitochondria contacts and have also removed the statement from the abstract (now reads: "Loss of TMEM24 results in hyper-accumulation of Ca^{2+} in the ER and in excess Ca^{2+} entry into mitochondria, with resulting impairment in glucose-stimulated ATP production.").

2. The main conclusion of this study is TMEM24 is a regulator of cellular Ca2+ homeostasis. Nevertheless, the data supporting this conclusion were derived only from TMEM24 KO MIN6 cells, and it is unclear how TMEM24 regulates ER and mitochondrial Ca2+ levels. At a minimum, the authors should rule out the possibility that abnormal Ca2+ levels in the ER and mitochondria is not a pleiotropic effect in TMEM24 KO cells. Does re-expression of TMEM24 rescue ER and mitochondria Ca2+ overload in TMEM24 KO MIN6 cells? Also, is ER and mitochondria Ca2+ overload observed in

© 2021. Published by The Company of Biologists under the terms of the Creative Commons Attribution License (https://creativecommons.org/licenses/by/4.0/).

TMEM24 knockdown MIN6 cells?

We show in the paper that the addition of thapsigargin under Ca2+-free conditions, i.e. where no store- operated Ca2+ influx occurs, causes a larger rise of cytosolic Ca2+ in TMEM24 KO cells, and that this response is normalized by the re-expression of TMEM24 (Fig. 3L). This rise of cytosolic Ca2+ reflects the release from ER. We have now complemented these experiments with experiments on wild type MIN6 cells where TMEM24 expression was transiently reduced by siRNA, and find that release of Ca2+ from the ER of these cells, either by CPA or charbachol, results in larger rises of cytosolic Ca2+ than in control cells (new Suppl Fig 2A, B). We also show that over-expression of wild type TMEM24 results in reduced CPA-induced release of Ca2+ from the ER (new Suppl Fig 2C). Together these experiments strengthen the conclusion that TMEM24 is a regulator of ER Ca2+ homeostasis.

Unfortunately, we were unable to perform rescue experiments in TMEM24 KO cells using the ER-localized Ca2+ sensor D4ER, since the excitation and emission properties of this FRET sensor was not compatible with either GFP or mCherry-tagged TMEM24 on our experimental setups (since the transfection efficiency of these cells is low, it is necessary to have a fluorescent tag on the protein to be expressed in order to select cells for analysis). For the mitochondrial Ca2+ measurements, we show in Fig. 4K and 4L that re- expression of wildtype TMEM24 in TMEM24 KO cells normalize the mitochondrial Ca2+ overload in response to depolarization.

3. This study claims that TMEM24 is not required for glucose-stimulated insulin secretion which is contrast to previous findings. Are the observed differences caused by the choice of cell lines or different experimental conditions? Another beta cell model should be tested in order to rule out that the finding in this study only applies to MIN6 cells.

We understand the reviewers concern that what we observe may be cell line specific rather than related to beta cell function. Nevertheless, we feel that the extent of work required to fully characterize another beta cell line is unrealistic. We could perform insulin secretion measurement in another beta cell line, but irrespective of the outcome of TMEM24 KO (no effect or suppressed secretion) we would still have to continue with full characterization of cytosolic and organellar Ca2+ homeostasis, cellular metabolism measurements, assessment of mitochondrial function and more in this cell line in order to be able to compare the results to the ones obtained from MIN6 cells. We believe that the MIN6 cells kept in our lab are highly functional (our cell line increase insulin secretion 400-500% in response to glucose challenge, which can be compared to the previous TMEM24 studies where secretion was increased 50-100% in both INS1 and MIN6 cells) and a good model of primary beta-cell function. One difference between our study and that of Pottekat et al (Cell reports, 2013) is that they measure insulin secretion from MIN6 pseudoislets, which are known to be more functional than MIN6 cells cultured as monolayers (PMID: 23604550), primarily through improved glucose oxidation and oxidative phosphorylation. We have therefore included dynamic insulin secretion measurements from wildtype and TMEM24 KO pseudo-islets, where we are also able to distinguish the two phases of insulin secretion. We found that whereas TMEM24 KO was without effect on the 1st phase of insulin secretion, it slightly impaired 2nd phase secretion (Fig. 2O). Such an impairment may have been masked in our 30 min static insulin secretion measurements performed on monolayer cultures (Fig. 2N). In the Lees et al paper (Science, 2017), they assay insulin secretion after 1 h incubation in high glucose, and under these conditions the relative proportion of insulin secreted during the second phase is much larger, which may explain why they observe a secretion defect and we do not. We now also more extensively discuss the differences in insulin secretion defects following TMEM24 KO in the manuscript (highlighted in yellow).

Minor concerns:

1. In some places, the descriptions in the figure legends and main texts are not consistent. For example: it says in the text that Ca2+ responses were measured using Cal520 but in the legend of Figure 2, Fluo4 was mentioned instead.

© 2021. Published by The Company of Biologists under the terms of the Creative Commons Attribution License (https://creativecommons.org/licenses/by/4.0/).

Thank you for noticing this. We have now made corrections to the text and in the figure legends (highlighted in yellow).

2. The figure legends and methods sections did not provide sufficient details. For example, in Figure 3O, the concentration of thapsigargin used was not specified. Also, the amount of time cells were treated with tharsigargin was not described.

This information has now been added to the figure legend "Average cytosolic Ca2+ concentration changes in wildtype (black), TMEM24 KO (blue) and TMEM24 KO with re-expression of TMEM24 (red) in response to 30 mM KCl alone (top row) or after 10 min treatment with 1μM thapsigargin (bottom row). Data are averages from 32-43 cells from 1 experiment per condition." We have also added additional details to figure legends and the methods section (highlighted in yellow). We have also made sure that concentrations of all other drugs are clearly presented in the figure legends (see markings in yellow).

3. The manuscript will benefit from careful scientific proof-reading. There are some mistakes in the text. For example, it says "….electrostatic interactions between the C-terminus and cationic lipids in the plasma membrane". It should be "negatively charged lipids" in the plasma membrane.

We thank the reviewer for the noticing this. The sentence has been corrected.

4. The authors showed that TMEM24 dissociation from ER-PM contacts can be triggered by carbachol stimulation after ER Ca2+ depletion or PMA addition. Based on these results, the authors claim that TMEM24 binding to the plasma membrane can be regulated by DAG. The authors did not directly demonstrate the involvement of DAG in regulating TMEM24 dissociation. Thus, the conclusion should be modified accordingly.

PMA is a DAG analogue that activates DAG-sensitive PKC isoforms and cause their interaction with the plasma membrane of beta cells in a Ca2+ independent manner (see e.g. PMID: 2656716). We showed that addition of PMA cause dissociation of TMEM24 from the plasma membrane (Fig. 1B, C). We now additionally show that this dissociation occurs without any change in the cytosolic Ca2+ concentration (new Suppl. Fig. 1A, B) and that the presence of PMA reduced the Ca2+-induced TMEM24 plasma membrane dissociation in permeabilized cells (effect is smaller since PMA induce dissociation by itself, showing that Ca2+ and PMA operate via the same mechanism) (new Suppl. Fig. 1H, I). Moreover, we now provide quantifications of TMEM24 plasma membrane dissociation in response to CPA and the combination of CPA and charbachol, and show that charbachol induce pronounced dissociation of TMEM24 under conditions when there are no changes in the cytosolic Ca2+ concentration (but DAG can still form in response to PLC activation, see e.g. Fig. 4B in PMID: 27226533). Together, we believe these experiments show that DAG, in addition to Ca2+, can induce the dissociation of TMEM24 from the plasma membrane.

<u>Second decision letter</u>

MS ID#: JOCES/2021/259073

MS TITLE: The endoplasmic reticulum-plasma membrane tethering protein TMEM24 is a regulator of cellular Ca2+ homeostasis

AUTHORS: Beichen Xie, Styliani Panagiotou, Jing Cen, Patrick Gilon, Peter Bergsten, and Olof Idevall-Hagren
ARTICLE TYPE: Research Article

I am happy to tell you that your manuscript has been accepted for publication in Journal of Cell Science, pending standard ethics checks. I appreciate your thoughtful responses to the reviewers' comments. The additional data and discussion strengthen the conclusions. Thank you for submitting this interesting work to JCS!

© 2021. Published by The Company of Biologists under the terms of the Creative Commons Attribution License (https://creativecommons.org/licenses/by/4.0/).
